# Vertebral Arteria Lusoria and Five Vessel Aortic Arch: A Rare Aortic Arch Branching Anomaly

**DOI:** 10.7759/cureus.67988

**Published:** 2024-08-28

**Authors:** Resham Singh, Vineeta Ojha, Sivasubramanian Ramakrishnan, Rushikesh Shukla, Sanjeev Kumar

**Affiliations:** 1 Cardiovascular Radiology and Endovascular Interventions, All India Institute of Medical Sciences, New Delhi, New Delhi, IND; 2 Cardiology, All India Institute of Medical Sciences, New Delhi, New Delhi, IND; 3 Radiology, Datta Meghe Institute of Higher Education and Research, Wardha, IND

**Keywords:** common carotid artery, brachiocephalic trunk, aortic arch, vertebral artery, ct angiography

## Abstract

Vertebral artery (VA) lusoria is an unusual variation of the VA origin and course, characterised by the direct origin of the right VA (RVA) from the aortic arch (AoA) instead of the right subclavian artery (RSCA). Generally, this condition remains asymptomatic and is diagnosed during computed tomography angiography (CTA) or catheter angiography performed for evaluation of other cardiac or extracardiac pathology. The surgeon and physician must be aware of this VA-origin anomaly before undergoing surgery or angiography, as injury to this vessel can be catastrophic and lead to torrential haemorrhage or brain stem infarction. This anomaly can occur in association with other branching anomalies, like the left VA originating from the arch and the bovine arch. We are reporting an unusual case of aberrant RVA in a five-vessel aortic arch We aim to highlight the importance of CTA in detecting these anomalies of origin and course and their clinical implications.

## Introduction

The vertebral arteria lusoria is one of the few congenital aberrations of the vertebral artery (VA), where it arises directly from the aortic arch (AoA) instead of the right subclavian artery (RSCA). Hyrtl first described this anomaly in 1859, where he found VA took its anomalous origin from the AoA and coursed behind the oesophagus and trachea before entering the cervical transverse foramen [1]. We represent a case of vertebral arteria lusoria coexisting with the direct origin of left VA (LVA) in a five-vessel aortic arch in a patient with tetralogy of Fallot (ToF), deciphered on computed tomography angiography (CTA).

## Case presentation

**Figure 1:**
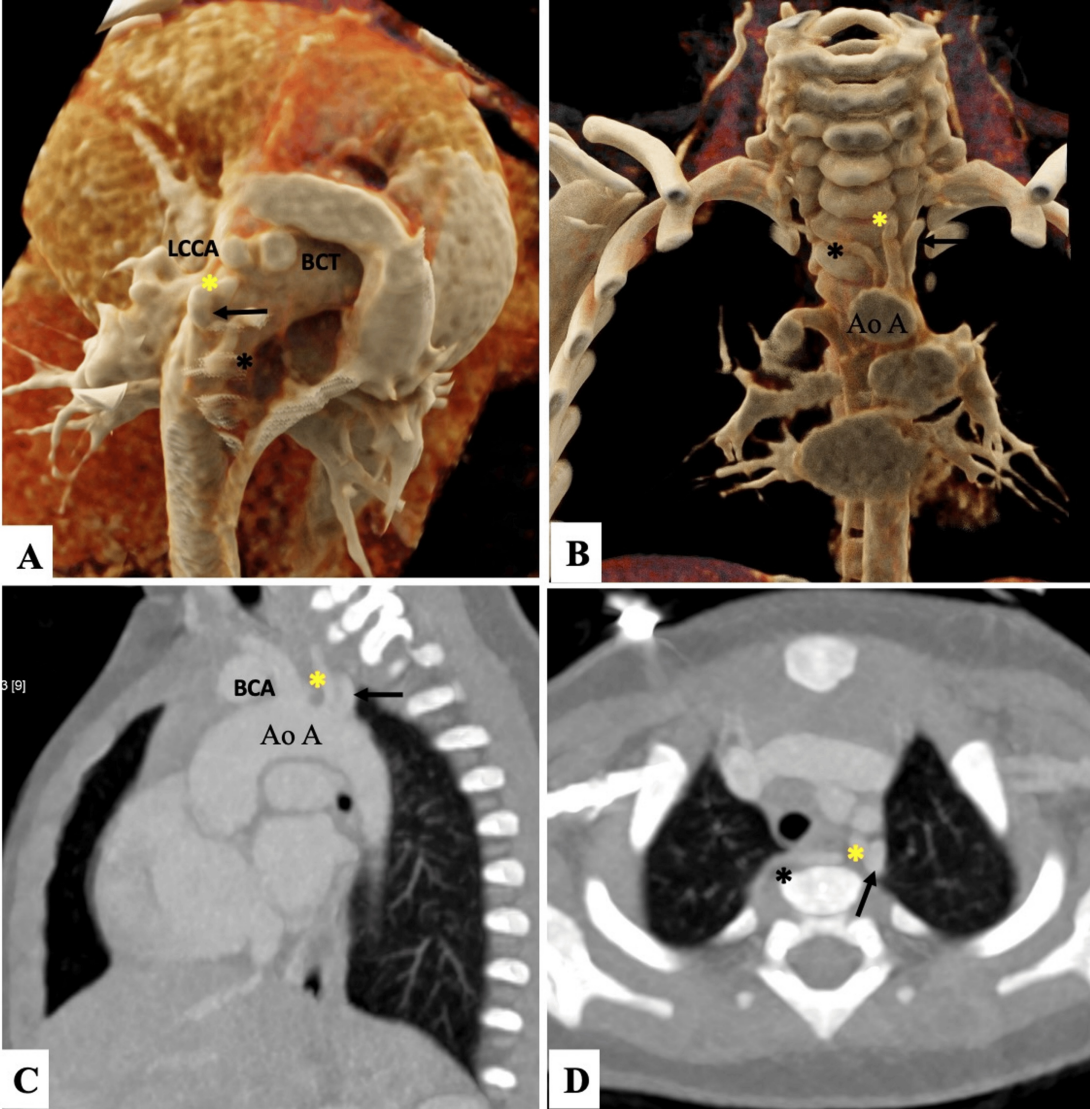
Cinematic volume rendered (A-B) and maximum intensity projection images (C-D) of rare aortic arch branching anomaly. Cinematic volume rendered (VRT) images (A,B) and sagittal oblique and axial maximum intensity projection (MIP) images (C,D) show five vessels aortic arch with the close origin of the IA and LCCA. The direct origin of the LVA (yellow asterisk, A-D) is seen between the left subclavian artery (LSCA) and LCCA (LSCA-straight black arrow in A-D). The aberrant origin of the RVA (black asterisk, A, B, D) is also seen from the right posterolateral aspect of the proximal descending thoracic aorta. The RVA has a retro-oesophageal course before having a normal transforaminal course through the cervical vertebrae, known as vertebral arteria lusoria.

A one-year-old child presented with bluish discolouration of the skin and failure to thrive since birth. Transthoracic echocardiography revealed the findings of ToF. A CTA was done for further evaluation of cardiac and extracardiac anatomy. The CTA validated the echocardiography findings. In addition, the patient had an AoA branching abnormality. The AoA was left-sided, with five vessels branching out. The innominate artery (IA) and left common carotid artery (LCCA) had a close origin from AoA (Figure 1A). The LVA originated directly from AoA between LCCA and left subclavian artery (LSCA) (yellow asterisk, Figure 1A-1D). The right VA (RVA) originated abnormally on the right posterolateral side of the descending thoracic aorta, just distal to the LSCA origin (black asterisk in Figure 1A, 1B, 1D). Before entering the transverse foramina of the C6 vertebra (black asterisk, Figure 1B, 1D), the RVA went backwards and forwards across the oesophagus between the upper thoracic spine and the oesophagus. It then went ventrally towards the right haemithorax (vertebral arteria lusoria).

## Discussion

Anomalous origin of VA occurs most frequently when they originate directly from the arch or aorta, with a prevalence ranging from 2.4% to 5.8% [2]. However, we can classify the extremely rare occurrence of aberrant origins of the RVA into three distinct categories: those directly originating from the aorta, those originating from the brachiocephalic or carotid arteries, and those with duplicated origins [3]. The VA is typically formed from the anastomoses of the seven cervical intersegmental arteries (ISAs). Except for the seventh internal thoracic artery (ITA), which is a component of the subclavian artery and serves as the source of VAs, all other ISAs gradually diminish in size. Moreover, in individuals with excellent health, the embryological right dorsal aorta typically undergoes natural regression. The close portion of the right fourth arch remains incorporated into the right SCA, whereas the distal half undergoes regression. As stated in the prior literature [2,4], the normal development of the cervical ISA's longitudinal anastomosis, the continued existence of the right dorsal aorta, and the degeneration of the right 4th AoA can all contribute to the presence of "vertebral arteria lusoria" in this case. The positioning and timing of these connections determine the final structure of the arch and the growth of its branches. Anomalous connection at any stage of the arch's embryonic development leads to the atypical creation of these vertebral arteries.

Documenting these unexpected abnormalities in the origin of VA, discovered incidentally due to an aberrant course, is crucial as they may raise the risk of dissection or aneurysms. Understanding these anatomical differences is crucial in preventing unintentional harm from medical interventions, which can result in a lack of blood supply to the brainstem during procedures like catheter angiography or cardiothoracic surgery.

## Conclusions

This case report illustrates an uncommon origin anomaly in the RVA. Its purpose is to emphasise the significance of CTA in identifying such rare anomalies and understanding their clinical implications.
